# Effects of Backfat Thickness on Oxidative Stress and Inflammation of Placenta in Large White Pigs

**DOI:** 10.3390/vetsci9060302

**Published:** 2022-06-19

**Authors:** Jian Hu, Peishi Yan

**Affiliations:** College of Animal Science and Technology, Nanjing Agricultural University, Nanjing 210095, China; hujian201381@163.com

**Keywords:** sows, backfat thickness, reproduction, lipid metabolism, oxidative stress, inflammation

## Abstract

The purpose of this study was to evaluate the impact of the backfat thickness of sows on reproductive performance and on lipid metabolism, oxidative stress, and inflammation. At farrowing, 60 sows were assigned to three groups: the low-backfat-thickness group (LBF, *n* = 20): sows’ backfat thickness was between 9 and 12 mm; the medium-backfat-thickness group (MBF, *n* = 20): sows’ backfat thickness was between 13 and 20 mm; and the high-backfat-thickness group (HBF, *n* = 20): sows’ backfat thickness was between 21 and 25 mm. Maternal and fetal blood and placental samples were collected. Compared with the LBF and HBF groups, the MBF group delivered a significantly greater number of live piglets than the LBF or HBF groups. The different backfat thicknesses of sows had different effects on the lipid-related hormones and adipokines of maternal and fetal serum and placenta. Sows with poor or excessive backfat displayed higher levels of oxidative stress and higher levels of pro-inflammatory cytokines. According to these data, the thickness of a sow’s backfat affects the characteristics of farrowing piglets and their lipid metabolism, as well as placental inflammation, maternal inflammation, and oxidative stress. A moderate backfat thickness (between 13 and 20 mm) was associated with greater reproductive performance in sows.

## 1. Introduction

The reproductive performance of sows is currently considered one of the most challenging subjects in the swine industry and is also the key to increasing the production profitability of the pig industry. The backfat thickness measurement serves as a useful tool for monitoring and improving the efficiency of farms [1]. In pigs, during gestation and lactation, an excessive loss of backfat has been significantly correlated with the number of stillborn piglets, the size and growth of litters, and prolonged weaning to the estrus interval in several studies [2,3]. Excessive backfat in sows is linked to changes in the decreasing volume of the abdominal cavity, which corresponds with the impact of placental and fetal development during late gestation [4]. A relationship has been established between backfat thickness and reproductive performance in sows; the molecular mechanisms connecting maternal backfat thickness to the characteristics of newborn piglets at farrowing as well as to maternal, placental, and fetal lipid metabolism remain elusive.

The maternal conditions are demonstrated to be closely related to the endocrine function of sows and piglets [5]. Pregnant sows are characterized by a high level of oxidative stress, which further influences the endocrine function of the fetoplacental unit [6,7]. Additionally, oxidative stress in placental tissue due to redox imbalance stimulates the generation of inflammatory mediators and inflammation, which leads to placental insufficiency [8]. Herein, we hypothesize that the thickness of the backfat of sows is related to placental oxidative stress and inflammation. Consequently, the purpose of this study was to assess the impact of backfat thickness on the characteristics of newborn piglets at farrowing and their lipid metabolism and other measures of inflammation and oxidative stress. Furthermore, the data collected were used to test our hypothesis about the correlation between backfat thickness in sows and placental oxidative stress and inflammation, as well as the correlation between sows and their piglets.

## 2. Materials and Methods

### 2.1. Experimental Animals 

The experiment was conducted at the Research Farm of Nanjing Agricultural University where there were no special provisions to control the environmental conditions of the experimental animals. A total of 60 large white sows (parity from 3–5) were chosen from a herd of 151, all housed in passively ventilated gestation stalls (2.20 m × 0.65 m) with concrete slatted floors. Sows had access to free water and were fed at 0900 and 1600 h daily with a standard grain-based diet (Table S1). The diet was purchased from Jiangsu Zhengchang Industrial Co. Ltd. Sows were fed formula diets that were designed to meet the nutrient requirements of pregnancy development by the NRC (2012). Sow backfat thickness was measured at 65 mm to the left side of the dorsal midline at the level of the last rib (known as the P2 point) using an ultrasonic scanner (Lean-meter, Renco, Minneapolis, MN, USA) at approximately day 110 of gestation [9]. Measuring units are given in millimeters. Based on backfat thickness, sows (*n* = 60) were assigned to three groups, a low-backfat-thickness group (LBF, *n* = 20): sows’ backfat thickness was between 9 and 12 mm; a medium-backfat-thickness group (MBF, *n* = 20): sows’ backfat thickness was between 13 and 20 mm; and a high-backfat-thickness group (HBF, *n* = 20): sows’ backfat thickness was between 21 and 25 mm. 

### 2.2. Data and Sample Collection

There are several indicators for measuring litter size at birth in pigs, including the number of piglets born, number of live piglets, mummified fetuses, stillborn piglets, litter weight at birth, average birth weight, and placenta weight. Placental efficiency was calculated by dividing piglet weight by placental weight, and stillbirth ratio was calculated by dividing stillborn piglets by the total litter of piglets. One piglet per litter (body weight close to the average weight of the litter) was selected at birth, and the corresponding placentas of selected piglets were collected. Samples were immediately collected from the maternal jugular vein and piglet umbilical cord after delivery. Samples of blood were collected in 5 mL sterile vacuum tubes (Greiner, Frickenhausen, Germany) and then centrifuged for 15 min at 4 °C at 3000× *g*. 

To match piglets with their placentas, placentas were collected immediately within 20 min of delivery according to the modified method suggested by Wilson et al. [10]. After the expulsion of the piglet, the umbilical cord was marked and tied with a silk line labeled with a tag. The birth weight and birth order of newborn piglets were noted. Placenta samples were excised from the maternal side of the placenta at 2 cm from the insertion point of the umbilical cord, and maternal blood and decidua were removed. After collection, placental tissues were washed with phosphate-buffered saline (PBS), minced with dissecting scissors and placed into 1.5 mL tubes. During collection, all samples were kept on ice and frozen at −80 °C. Biochemical and gene expression analysis of the placental samples was performed on individual samples by group. A total of 60 placental samples were examined (20 per group) in this study.

### 2.3. Biochemical Analysis

For biochemical analysis, approximately 0.5 g of each placenta tissue sample was homogenized in 5 mL 0.85% chilled normal saline. Supernatants were further centrifuged for 10 min at 4 °C at 3000× *g* and were then used for analysis. Commercial ELISA kits were purchased from RD systems (Minneapolis, MN, USA) and were used to detect the contents of insulin (INS), leptin (LEP), adiponectin (ADP), tumor necrosis factor-alpha (TNF-α), cytokine interleukin 6 (IL-6), cytokine interleukin 8 (IL-8), and cytokine interleukin 1β (IL-1β) in sow serum, piglet cord serum, and placenta homogenate. All samples were measured in a single assay, and all intra-assay and inter-assay CVs were <10% and <15%, respectively. Low-density lipoprotein cholesterol (LDL-c), high-density lipoprotein cholesterol (HDL-c), and glucose (GLU) concentrations in maternal and fetal serum were analyzed with a Unicel 36 DX600 Synchron (Beckman Coulter, Mississauga, ON, Canada). The commercial kits used for these measurements included the total triglyceride (TG), the free fatty acids (FFA), the cholesterol (CHO), the malondialdehyde (MDA), the inhibition of hydroxyl radical (OH), the total antioxidant capacity (T-AOC), and the glutathione peroxidase (GSH-Px), superoxide dismutase (SOD), and catalase (CAT) activities.

### 2.4. Gene Expression Analysis of Placenta

Total RNA from placenta samples was processed using TRIZOL reagent (Sigma-Aldrich, St. Louis, MO, USA) as directed by the manufacturer. Quantification of total RNA was performed using a NanoDrop^®^ ND-1000 Spectrophotometer. About 1 µg of total RNA was converted into cDNA using the reverse transcription cDNA kit (Thermo Fisher Scientific, Waltham, MA, USA) as per the manufacturer’s instructions. Real-time qPCR was performed with 1 µL cDNA in a total volume of 20 µL with SYBR Green PCR master mix (Invitrogen, Carlsbad, CA, USA) using 7900HT Fast Real-Time PCR System (Life Technology, Carlsbad, CA, USA). The primer sequences are summarized in Table S2. 

The 20 µL reaction system consisted of the cDNA template, forward and reverse primers, and 1× SYBR Green PCR Master Mix. The real-time qPCR running protocol was set as follows: 10 min at 95 °C for initial denaturation, followed by 15 s at 95 °C for denaturation, 10 s at the primer-specific temperature (Table S2) for annealing, then 30 s at 72 °C for an extension. All real-time qPCR data were normalized through reference genes, β-acting, and glyceraldehyde-3-phosphate dehydrogenase (GAPDH). Each PCR reaction was performed in triplicate. To confirm the specificity of the amplification, a dissociation procedure was used to generate melting curves. The relative expression of target genes was calculated via the 2–ΔΔCT method.

### 2.5. Statistical Analysis

IBM SPSS 20.0 (IBM SPSS Statistics, Armonk, NY, USA) was used for statistical analyses. For the results of the reproductive performance, biochemical indexes, and real-time qPCR, values are displayed as the mean ± S.E.M. Multiple group comparisons were carried out by one-way ANOVA, followed by a Student–Newman–Keuls test. Regression analyses were conducted using the Proc MIXED procedure in SAS version 9.2 (SAS Institute, Cary, NC, USA). The categorized backfat thickness at 110 d of gestation was specified as a fixed effect. The response variables were related measurements, including lipid-related hormones, oxidative stress, and inflammation factors. Differences were considered significant when *p* < 0.05 and as a tendency when 0.05 ≤ *p* ≤ 0.10.

## 3. Results

### 3.1. Maternal, Placental, Neonatal Characteristics

Compared to the LBF and HBF groups, the MBF group had more piglets per litter and more piglets born alive per litter (*p* < 0.05, Table 1) than the LBF and HBF groups, but the MBF and HBF groups did not show any differences (*p* > 0.05) (Table 1). In the MBF group, the litter birth weight was higher (*p* < 0.05) than in the LBF and HBF groups. Stillborn piglets were significantly fewer per litter (*p* < 0.05) in the MBF group, but the values were not significantly different from those in the LBF and HBF groups (*p* > 0.05). Of the three groups, the LBF group had the highest stillbirth rate, whereas the MBF group had the lowest number. The average birth weight of the HBF group was similar to that of the LBF and MBF groups, of which the MBF group was the heaviest (*p* < 0.05). Mummified fetuses per litter and placenta weight did not differ between the three groups (*p* > 0.05). 

### 3.2. Lipids and Lipid-Related Hormones and Adipokines in Maternal and Fetal Serum and Placenta Homogenate

In the MBF group, the TG concentration of the sow serum and placenta homogenate was (*p* < 0.05) lower than in the LBF and HBF groups, whereas the piglet cord serum TG concentrations were higher (*p* < 0.05) than in the HBF group (Table 2). The sow serum concentrations of HDL were higher (*p* < 0.05) in the MBF group than in the LBF and HBF groups, but the values of piglet cord serum did not differ (*p* > 0.05) among the three groups. In addition, the LEP level of piglet cord serum was significantly and linearly correlated with backfat thickness (*p* = 0.01; *p* < 0.01). 

In the LBF group, the concentrations of INS and LEP of the placenta homogenate were lower (*p* < 0.05) than in the HBF group, and the concentrations did not differ significantly between the MBF or HBF groups and the LBF group. The correlated analysis results showed that TG concentrations in the piglet cord serum and placenta homogenate were significantly associated with backfat thickness (*p* < 0.01, *p* = 0.04, respectively; Table 2). There was a linear increase in the CHOL concentrations of maternal blood with increased backfat thickness (*p* = 0.04). Additionally, a tendency for a linear increase in the NEFA concentrations of the placenta homogenate was observed with increased backfat thickness (*p* = 0.03).

### 3.3. Oxidant/Antioxidant Status in Maternal and Fetal Serum and Placenta Homogenate 

In the LBF and MBF groups, the MDA concentration of sow serum, as well as in the piglet cord serum and placenta homogenate, was lower than in the HBF group (*p* > 0.05), and the changes in sow and piglet cord serum exhibited a linear increase as the backfat thickness increased (*p* < 0.01, *p* < 0.01, respectively; Table 3). In the MBF group, the SOD concentrations in the sow serum and placenta homogenate were higher (*p* < 0.05) than in the LBF or HBF groups, and the changes in the placenta homogenate exhibited a linear decrease alongside backfat thickness (*p* = 0.02). The OH· inhibition of the sow and piglet cord serum was higher (*p* < 0.05) in the HBF than in the LBF and HBF groups, and the OH· inhibition of the placenta homogenate was no different (*p* > 0.05) in the three groups. In addition, there was a linear decrease in the OH· inhibition and GSH-Px concentration in the piglet cord serum with increased backfat thickness (*p* < 0.01, *p* < 0.01, respectively). 

In the MBF group, the CAT concentrations in the piglet cord serum and placenta homogenate were lower (*p* < 0.05) than in the LBF or HBF groups. The sow serum concentration of TAC was greater (*p* < 0.05) in the MBF than in the LBF and HBF groups, and the TAC concentration of the placenta homogenate in the LBF and MBF groups was increased (*p* < 0.05) compared with the HBF group. There was a linear increase in the CAT concentration in the piglet cord serum with increased backfat thickness (*p* = 0.04). 

### 3.4. Pro-Inflammatory Cytokines in Maternal and Fetal Serum and Placenta Homogenate

In the MBF group, the tumor necrosis factor-α (TNF-α) concentration in sow and piglet cord serum was not significantly different from that in the LBF and HBF groups, but the HBF group had a higher concentration of sow serum (*p* < 0.05) and a lower concentration of cord serum in piglets (*p* < 0.05) than in the LBF group (Table 4). No difference (*p* > 0.05) in the TNF-α concentration in the placenta homogenate, nor the IL-6 concentration in the placenta homogenate, was found between the MBF or HBF groups and the LBF group, whereas the concentrations of TNF-α and IL-6 in the placenta homogenate were lower (*p* < 0.05) in the MBF than the HBF group. In the LBF and MBF groups, the IL-6 concentration of the sow serum was reduced (*p* < 0.05) compared with the HBF group; however, there were higher IL-6 concentrations (*p* < 0.05) in the placenta homogenate than in the HBF group. The correlated analysis results showed that the TNF-α and IL-6 concentrations in the sow serum were significantly associated with backfat thickness (*p* < 0.01, *p* = 0.02, respectively). In addition, there was a linear decrease in the TNF-α concentration of the piglet cord serum with increased backfat thickness (*p* = 0.04). 

### 3.5. Inflammatory Genes’ Expression in Placenta 

**Figure 1:**
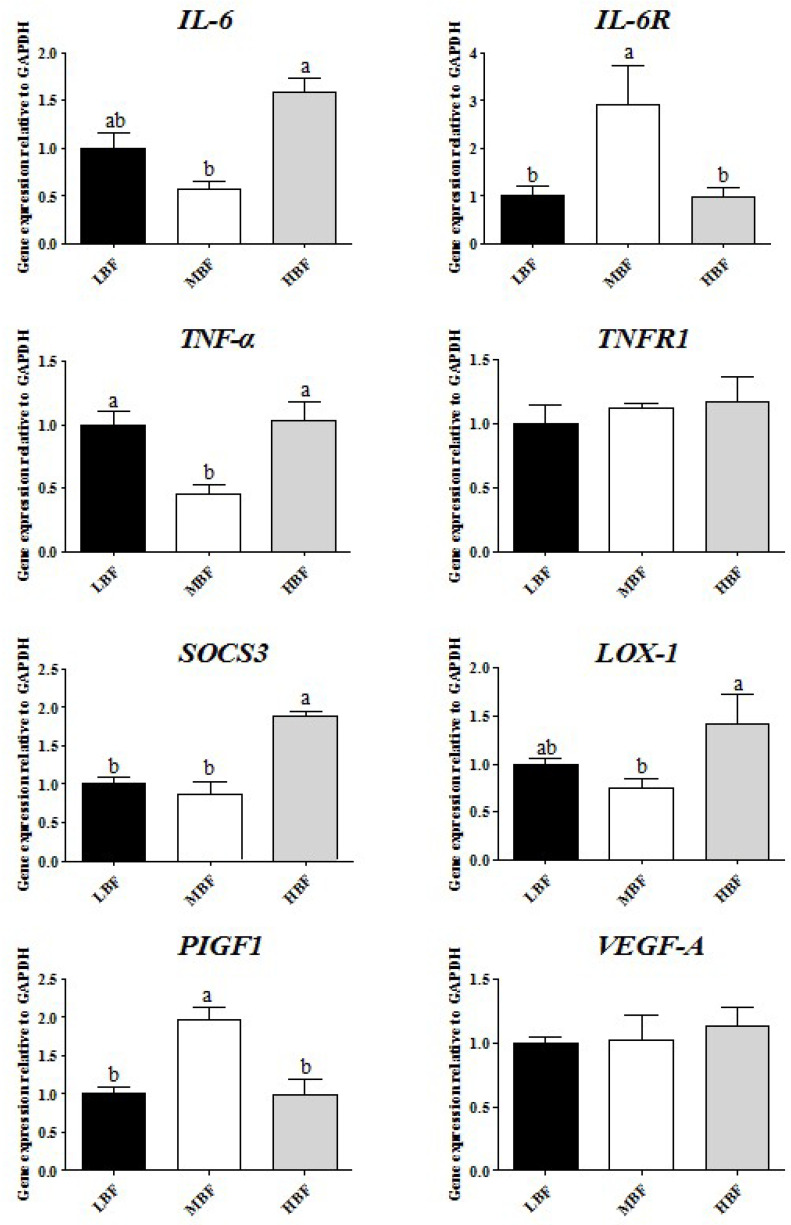
The mRNA levels of inflammatory genes in the placenta according to backfat thickness. Values are mean ± S.E.M. A difference in letter between columns is significant at *p* < 0.05.

The mRNA expressions of IL-6 and lectin-like oxidized LDL receptor-1 (LOX-1) were significantly lower in the MBF group than in the HBF group and were similar to those in the LBF group (Figure 1). Compared with the MBF group, however, the mRNA expression of the cytokine interleukin 6 receptor (IL-6R) was reduced in the LBF and HBF groups. The MBF groups expressed lower levels of TNF-α mRNA (*p* < 0.05) than the LBF and HBF groups, but the values between the LBF and HBF groups did not differ significantly (*p* > 0.05). In the LBF and HBF groups, suppressors of cytokine signaling 3 (SOCS3) mRNA expression were decreased (*p* < 0.05) compared with the HBF group, but no significant difference (*p* > 0.05) was found between the LBF and MBF groups. The MBF group showed a higher level of mRNA expression of placental growth factor-1 (PIGF-1) than the LBF and HBF groups (*p* < 0.05), but no significant difference (*p* > 0.05) was found between the three groups. Among the groups, there were no significant differences in the mRNA levels of tumor necrosis factor receptor-1 (TNF-R1) and vascular endothelial growth factor-a (VEGF-A) (*p* > 0.05).

## 4. Discussion

Previous studies have established the relationship between backfat thickness and reproductive performance in sows, and they mainly focused on the excess backfat which caused difficulties that influenced fetuses. However, the effects of the backfat thickness of sows on placental inflammation, oxidative stress, and placental lipid concentrations remain to be elucidated. According to the current study, the concentrations of lipids involved in the metabolism and pro-inflammatory cytokines; the oxidant/antioxidant statuses of the sow serum, piglet cord serum, and placenta homogenate; and the relative mRNA levels of inflammatory genes in the placenta were measured to assess how the thickness of backfat influenced reproductive performance, lipid metabolism, antioxidant status, and inflammation.

### 4.1. Effects of Backfat Thickness on Reproductive Performance

When sows have excess backfat during gestation, they tend to have problems farrowing, more stillborn piglets, and other reproductive performance disorders [11]. Moreover, piglets weaned from sows with lower backfat thickness are less numerous [12]. Consistent with previous research [13,14], piglet births per litter, as well as piglets born alive, were significantly higher in the MBF group than in LBF or HBF groups. Additionally, the MBF group had a significantly lower number of stillbirths per litter than either the LBF or HBF groups, but these values did not differ between the groups [14]. Excessive backfat of sows is associated with changes in the decreasing volume of the abdominal cavity, which could impact placental and fetal development [4]. The results of our study agree with previous research showing that a high backfat thickness in sows could cause many stillborn piglets [3]. There could also be a problem with fat deposition blocking the birth canal, which results in difficulties during parturition. According to this study, sows with a low backfat thickness had the highest rate of stillbirth, whereas the smallest litters had the lowest birth weights. The thin sows had low energy storage, which failed to regulate their normal physiological processes and maintain pregnancies [15]. A previous study has also shown that the placenta of neonates with a low birth weight is susceptible to oxidative damage [16]. Thus, sows with low backfat during pregnancy possess poor reproductive indices. A lower placental efficiency could relate to intrauterine growth retardation, and small-for-gestational-age offspring [17] and is also positively related to litter size [18]. In the present study, the MBF group had a significantly higher placental efficiency than the LBF and HBF groups. As reviewed by König et al. (2020), the placental efficiency in crossbred Danish genetic sows is higher than that in purebred German Saddleback sows, suggesting that this pattern contributes to a higher fecundity in breeds [19]. These observations indicate that maintaining moderate backfat thickness in sows is extremely important for optimizing fertility.

### 4.2. Effects of Backfat Thickness on Lipid-Related Hormones and Adipokines

An important role of lipid metabolism can be found in pregnancy initiation and development [20]. Fetal lipids, fetal growth, and fat mass are related to maternal serum TG and FFA [21]. In this study, a significantly lower concentration of TG in the MBF group was observed compared to the LBF and/or HBF groups, whereas the piglet cord serum TG concentration was significantly greater than in the HBF group. The above results indicated that sows with suitable backfat can support more TG for fetal lipogenesis. A higher level of the FFA of sow serum and placenta homogenate was found in the HBF group than in the LBF and/or MBF groups, but the piglet cord serum FFA was the same in all three groups. In our study, we found that higher concentrations of FFA were redistributed by the placenta and were not transferred to the fetus by simple diffusion. In the past, low blood HDL-c levels have been linked to pre-eclampsia and gestational diabetes in pregnant women [22]. In our study, the MBF group had significantly higher HDL-c levels in the sow serum than both the LBF and HBF groups. Thus, we speculate that sows with excess or poor backfat can experience negative pregnancy outcomes or can produce preterm births and low-birth-weight fetuses.

There has been evidence that placental nutrient transporters are stimulated by maternal hormones, including INS, LEP, and ADP [23]. It has been shown that obesity in sows leads to higher maternal plasma concentrations of INS and lower plasma levels of ADP in late pregnancy. Consistent with a previous study, higher values of INS in the sow serum and placenta homogenate were found in the HBF group compared to the LBF and/or MBF groups, but in contrast, the ADP levels of the sow serum and placenta homogenate were the lowest in the HBF group. A previous study indicated that a significant linear relationship between backfat thickness and plasma LEP was found [24]. Similarly, our work showed higher LEP levels in the piglet cord serum and placenta homogenate in the HBF groups than in the LBF or MBF group. However, the LEP level in the sow serum in the LBF groups was the highest among the three groups. The response to energy restriction in LEP is regulated by changes in adipocyte glucose and lipid metabolism, i.e., decreased glucose uptake and metabolism and increased lipolysis.

### 4.3. Effects of Backfat Thickness on Oxidant/Antioxidant Status and Pro-Inflammatory Cytokines 

We have found a complex variation of cytokines related to inflammatory and oxidative stress responses with different backfat thickness, especially between the HBF group and the MBF group. Compared with the LBF and the MBF groups, the MDA concentrations in the sow serum, piglet cord serum, and placenta homogenate were higher in the HBF groups. Similarly, increased oxidative stress and expression of pro-inflammation cytokines in the placenta are linked to excessive backfat thickness in sows [25]. This suggests that sows with excess backfat would experience oxidative stress. Additionally, sows in the MBF group had higher SOD concentrations in their serum and placenta homogenate than in the LBF or HBF groups. The inhibition of placental and maternal SOD observed here may be a critical factor for negative outcomes such as fetal death and mummified fetuses, which have greater incidence in sows with excess backfat [4]. It has been shown by a previous study that a positive correlation exists between maternal CAT and babies’ birth weights [26]. Consistent with the above study, the CAT concentrations in the sow serum were the highest in the MBF group. Previous research has indicated that increasing obesity correlates with increased ROS, such as OH· and hydrogen peroxide [27]. Moreover, the OH· inhibition and T-AOC level also represent the ability of the antioxidant system. Our results showed that sows with excess backfat had lower levels of OH· inhibition and T-AOC. The measurements of the pro-inflammatory cytokines in the placenta and maternal blood (TNF-α and IL-6) were primarily affected by oxidative stress. In a previous study, it was shown that TNF-α and IL-6 were associated with obesity or insulin resistance [28]. As part of this study, the TNF-α and IL-6 levels in maternal serum and placenta revealed a uniform trend toward being similarly increased in the HBF groups. Notably, the levels of TNF-α and IL-6 in the piglet cord serum were reduced in the HBF groups. Thus, we speculated that maternal obesity may amplify inflammation by increasing the expression of maternal and placental pro-inflammatory cytokines. However, the placenta plays a barrier role, inhibiting the inflammation of fetuses in this process [29].

### 4.4. Effects of Backfat Thickness on Inflammatory Genes’ Expression in Placenta

Pregnancy is characterized by a high level of oxidative stress, which further increases placental oxidative stress [6,30]. Oxidative stress in placental tissue due to a redox imbalance is causally associated with inflammation and inflammatory mediators [8]. We found that the expression of IL-6 and TNF-α mRNAs in the placenta was similar to the metabolites’ expression in the placenta homogenate, and the expression level was lower in sows with high backfat thickness. Compared with the MBF group, however, the mRNA expression of IL-6R was reduced in the LBF and HBF groups. The results of a previous study showed that a low IL-6R expression by target cells could preclude an IL-6-mediated protective response through the classical signaling pathway [31]. The SOCS3 gene is a negative feedback regulator of cytokine signaling. In particular, it inhibits insulin signaling by inhibiting leptin and downstream events. A previous study has shown that an increased expression of SOCS was found in obese subjects, which could cause preterm birth [32]. In our study, we also found that SOCS mRNA was expressed at the highest levels in the HBF group. LXA-1, one of the scavenger receptors, is recognized as a major ligand oxidized by low-density lipoprotein. It has been reported that the upregulation of LOX-1 in the placental tissues was associated with oxidative stress and inflammation [33]. LOX-1 mRNA expression was increased in the HBF group. These results further indicate that sows with excess backfat can experience increased oxidative stress and inflammation. PGF1 belongs to the family of vascular endothelial growth factors (VEGFs), which play an important role in placental angiogenesis. As demonstrated in a previous study, a reduction in PlGF1 expression was observed in gestational hypertensive disorders, small-for-gestational-age infants, as well as pre-term infants [34]. Thus, we speculate that sows with excess or lacking backfat could experience placental insufficiency.

## 5. Conclusions

In the present study, it was found that maternal backfat thickness influences the characteristics of newborn piglets at farrowing and as well as lipid metabolism, maternal and placental inflammation, and oxidative stress. Our findings also show that excess or poor backfat of sows was associated with a higher status of oxidative stress and a greater expression of pro-inflammatory cytokines. Additionally, sows with a moderate backfat thickness (between 13 and 20 mm) had greater reproductive performance.

## Figures and Tables

**Table 1:** Characteristics of sows and newborn piglets at farrowing ^1^.

Characteristics	Group LBF	Group MBF	Group HBF	SEM	*p*-Value
Litter size at birth in sows					
Total number of piglets born per litter (sow)	13.80 ^b^	15.14 ^a^	13.10 ^b^	0.29	0.04
Number of piglets born alive per litter (sow)	11.25 ^b^	13.01 ^a^	11.46 ^b^	0.25	0.04
Stillbirth piglets per litter (sow)	3.00 ^a^	1.51 ^b^	2.30 ^a^	0.15	0.03
Mummified fetuses per litter (sow)	0.31	0.27	0.29	0.06	0.23
Stillbirth ratio (%)	21.73%	9.97%	17.56%		
Litter birth weight (kg)	16.60 ^b^	19.65 ^a^	17.17 ^b^	0.40	<0.01
Average birth weight (kg)	1.35 ^b^	1.48 ^a^	1.42 ^ab^	0.02	0.04
Placenta					
Placenta weight (kg)	2.73	3.20	3.10	0.17	0.12
Placental efficiency	5.16 ^b^	6.14 ^a^	5.22 ^b^	0.17	0.03

^1^ All values are mean with pooled SEMs. In rows with different superscript letters, the values differ significantly (*p* < 0.05).

**Table 2:** The concentrations of lipids, lipid-related hormones, and adipokines in sow serum, piglet cord serum, and placenta homogenate ^1^.

Characteristic	Group LBF	Group MBF	Group HBF	SEM	*p*-Value	
					Q ^2^	L ^3^
Sow serum						
GLU (mmol·L^−1^)	3.98	4.13	3.85	0.12	0.63	0.49
TG (mmol·L^−1^)	0.94 ^a^	0.60 ^b^	0.99 ^a^	0.06	<0.01	0.07
FFA (mmol·L^−1^)	0.14 ^ab^	0.11 ^b^	0.16 ^a^	0.01	0.05	0.07
CHO (mmol·L^−1^)	6.23	6.03	6.88	0.20	0.26	0.04
HDL-c (mmol·L^−1^)	1.09 ^b^	1.27 ^a^	1.00 ^b^	0.03	0.03	0.45
LDL-c (mmol·L^−1^)	4.03	3.87	4.39	0.15	0.06	0.21
INS (mIU·L^−1^)	20.28	22.50	25.49	1.70	0.51	0.32
LEP (ng·mL^−1^)	18.34 ^a^	13.76 ^ab^	9.69 ^b^	1.10	0.04	0.08
ADP (mg·L^−1^)	8.14 ^ab^	13.64 ^a^	7.42 ^b^	1.32	0.05	0.37
Piglet cord serum						
GLU (mmol·L^−1^)	3.70	4.16	3.85	0.12	0.63	0.13
TG (mmol·L^−1^)	0.13 ^ab^	0.14 ^a^	0.10 ^b^	0.01	0.03	<0.01
FFA (mmol·L^−1^)	0.12	0.11	0.12	0.01	0.74	0.35
CHO (mmol·L^−1^)	1.04	1.19	1.20	0.05	0.57	0.17
HDL-c (mmol·L^−1^)	0.54	0.52	0.45	0.02	0.16	0.06
LDL-c (mmol·L^−1^)	0.11	0.18	0.16	0.03	0.79	0.33
INS (mIU·L^−1^)	25.10	24.41	22.19	0.89	0.09	0.21
LEP (ng·mL^−1^)	3.53 ^b^	6.15 ^a^	6.41 ^a^	0.35	0.01	<0.01
ADP (mg·L^−1^)	9.11	8.50	6.95	0.32	0.13	0.10
Placenta homogenate						
TG (mg·g^−1^ protein)	1.83 ^ab^	1.64 ^b^	2.23 ^a^	0.10	0.02	0.04
FFA (mg·g^−1^ protein)	1.18 ^b^	1.21 ^b^	1.64 ^a^	0.10	0.05	0.03
CHO (mg·g^−1^ protein)	0.92	0.80	1.08	0.06	0.17	0.11
INS (mIU·g^−1^ protein)	21.25 ^ab^	17.79 ^b^	22.70 ^a^	0.87	0.03	0.19
LEP (ug·g^−1^ protein)	4.64 ^ab^	3.87 ^b^	5.00 ^a^	0.18	0.03	0.167
ADP (mg·g^−1^ protein)	5.02 ^ab^	5.23 ^a^	4.16 ^b^	0.19	0.05	0.281

^1^ All values are mean with pooled SEMs. In rows with different superscript letters, the values differ significantly (*p* < 0.05); ^2^ Q: Quadratic discriminant analysis; ^3^ L: Linear discriminant analysis.

**Table 3:** Oxidant/antioxidant status in sow serum, piglet cord serum, and placenta homogenate ^1^.

Characteristic	Group LBF	Group MBF	Group HBF	SEM	*p*-Value	
					Q ^2^	L ^3^
Sow serum						
MDA (nmol·mL^−1^)	3.78 ^b^	3.35 ^b^	5.29 ^a^	0.30	<0.01	<0.01
SOD (U·mL^−1^)	39.06 ^b^	50.93 ^a^	40.96 ^b^	1.41	<0.01	0.33
OH· inhibition (U·mL^−1^)	41.66 ^a^	45.64 ^a^	34.75 ^b^	2.30	<0.01	0.25
GSH-Px (U·mL^−1^)	238.89	203.70	199.37	11.25	0.48	0.33
CAT (U·mL^−1^)	6.28 ^b^	6.97 ^a^	6.70 ^ab^	0.36	0.04	0.46
T-AOC (U·mL^−1^)	2.78 ^b^	4.54 ^a^	2.56 ^b^	0.42	0.03	0.15
Piglet cord serum						
MDA (nmol·mL^−1^)	1.84 ^b^	1.34 ^b^	2.41 ^a^	0.21	0.03	<0.01
SOD (U·mL^−1^)	18.86	19.95	20.24	0.58	0.06	0.48
OH· inhibition (U·mL^−1^)	46.67 ^a^	45.87 ^a^	38.45 ^b^	1.65	<0.01	<0.01
GSH-Px (U·mL^−1^)	106.67	90.37	87.62	7.4	0.16	<0.01
CAT (U·mL^−1^)	4.01 ^a^	3.18 ^b^	4.19 ^a^	0.25	0.04	0.04
T-AOC (U·mL^−1^)	2.28	2.87	2.42	0.19	0.45	0.36
Placenta homogenate						
MDA (nmol·mg^−1^ protein)	2.75 ^b^	3.16 ^b^	5.49 ^a^	0.38	<0.01	0.15
SOD (U·mg^−1^ protein)	11.99 ^ab^	12.83 ^a^	10.73 ^b^	0.45	0.04	0.02
OH· inhibition (U·mg^−1^ protein)	18.30	21.22	19.15	0.79	0.32	0.28
GSH-Px (U·mg^−1^ protein)	124.07	113.35	128.80	3.99	0.21	0.13
CAT (U·mg^−1^ protein)	5.78 ^b^	5.75 ^b^	7.12 ^a^	0.38	0.03	0.24
T-AOC (U·mg^−1^ protein)	0.95 ^a^	0.94 ^a^	0.65 ^b^	0.14	0.02	0.34

^1^ All values are mean with pooled SEMs. In rows with different superscript letters, the values differ significantly (*p* < 0.05); ^2^ Q: Quadratic discriminant analysis; ^3^ L: Linear discriminant analysis.

**Table 4:** The concentration of pro-inflammatory cytokines in sow serum, piglet cord serum, and placenta homogenate ^1^.

Characteristic	Group LBF	Group MBF	Group HBF	SEM	*p*-Value	
					Q ^2^	L ^3^
Sow serum						
TNF-α (ng·L^−1^)	57.85 ^b^	72.26 ^ab^	90.57 ^a^	5.02	0.04	<0.01
IL-6 (ng·L^−1^)	149.10 ^b^	198.45 ^b^	267.03 ^a^	12.55	0.03	0.02
IL-8 (ng·L^−1^)	76.86	65.34	81.90	4.33	0.29	0.24
IL-1β (ng·L^−1^)	46.83	61.34	65.90	3.33	0.44	0.28
Piglet cord serum						
TNF-α (ng·L^−1^)	73.84 ^a^	65.42 ^ab^	58.89 ^b^	1.99	0.03	0.04
IL-6 (ng·L^−1^)	111.82 ^a^	106.85 ^a^	98.67 ^b^	4.20	0.04	0.10
IL-8 (ng·L^−1^)	56.83	60.34	61.90	1.33	0.20	0.25
IL-1β (ng·L^−1^)	36.63	41.34	50.91	3.43	0.07	0.08
Placenta homogenate						
TNF-α (ng·g^−1^ protein)	22.39 ^ab^	21.10 ^b^	25.17 ^a^	0.77	0.04	0.20
IL-6 (ng·g^−1^ protein)	65.03 ^ab^	58.85 ^b^	75.11 ^a^	3.16	0.04	0.12
IL-8 (ng·g^−1^ protein)	76.84	68.64	79.90	3.63	0.24	0.19
IL-1β (ng·g^−1^ protein)	22.69 ^ab^	20.39 ^b^	34.31 ^a^	2.66	0.05	0.07

^1^ All values are mean with pooled SEMs. In rows with different superscript letters, the values differ significantly (*p* < 0.05); ^2^ Q: Quadratic discriminant analysis; ^3^ L: Linear discriminant analysis.

## Data Availability

Data are contained within the article or Supplementary Materials.

## Supplementary Materials

The following supporting information can be downloaded at: https://www.mdpi.com/article/10.3390/vetsci9060302/s1, Table S1: Ingredient and nutrient composition of the experimental diets; Table S2: Primer sequences for quantitative real-time polymerase chain reaction.
