# Supplementary material for: Effects of Backfat Thickness on Oxidative Stress and Inflammation of Placenta in Large White Pigs
Source: Vet Sci. 2022 Jun 19;9(6):302. doi: 10.3390/vetsci9060302 (PMC9230826; doi:10.3390/vetsci9060302)
Supplement: Supplementary file 1 [file vetsci-09-00302-s001.zip › vetsci-1738240-supplementary.pdf]

Table S1. Ingredient and nutrient composition of the experimental diets.

Feed Composition	Ratio
**Ingredients (%)**	
Corn	58.00
Soybean meal (43%)	18.00
Extruded soybean	6.00
Imported fish meal	3.50
Soybean oil	2.00
Sugar	2.50
Bran	6.00
Premix[1]	4.00
**Nutrient levels (%)**	
Crude protein	18.51
Crude fa	2.83
Crude fiber	2.77
Lys	0.99
Met + Cys	0.58
Arg	1.25
Ca (%)	0.40
P (%)	0.40
Na (%)	0.33
Ka (%)	0.29

[1] Vitamin and mineral premix supplied per kg diet: 9,600 IU vitamin A, 1,800 IU vitamin D_3, 24 mg vitamin E, 1.5 mg vitamin B_1, 12 mg vitamin B_2, 2.4 mg vitamin B_6, 0.045 mg vitamin B_{12}, 1.5 mg vitamin K_3, 24 mg pantothenic acid, 45 mg niacin, 0.09 mg biotin, 0.39 mg folic acid, 7.2 mg ethoxyquin, 167.58 mg Fe ($FeSO_4$), 15.23 mg Cu ($CuSO_4$; H2O), 153.09 mg Zn ($ZnSO_4$), 50.31 mg Mn (MnO_2), 0.9 mg I ($Ca(IO)_3$), 0.45 mg Se (Na_2SeO_3).

Table S2. Primer sequences for quantitative real-time polymerase chain reaction.

Gene	GenBank Accession no.	Primer sequences (5-3)		Annealing temperature (°C)	Length (bp)
		Forward	Reverse		
IL-6	NC_010451.3	AGTCCAGTCGCCTTCTCCCT	CAGAGATTTTGCCGAGGATG	61	87
IL-6R	NM_214403.1	GACCCTATGCGTCGCCAACA	TTGCCAGGTGACACTGAGCC	63	78
TNF-α	NC_010449.4	TTCTGCCTACTGCACTTCGA	GGCTTTGACATTGGCTACAA	58	128
TNFR1	NC_010447.4	GAACGCAGACTGCAAGAA	CTAAGCCAACGAAGAGGAA G	61	216
SOCS3	NC_010454.4	ATGGTCACCCACAGCAAGTT	AATCCGCTCTCCTGCAGCTT	60	128
LOX-1	NC_010447.4	TGCTCCACTCCCTCATTCTT	ACCAGTAATCCCAGGCACA G	60	111
PIGF1	NC_010445.4	TGTCCTTCTGAGTCGCTGTA GT	GCATAGTGATGTTGGCTGTC TT	59	110
VEGF-A	NC_010449.5	CAACGACGAAGGTCTGGAG TG	GCCTCGCTCTATCTTTCTTTG G	61	124
GAPDH	NC_010447.4	CGTCAAGCTCATTTCCTGGT	TGGGATGGAAACTGGAAGT C	59	210
